# MMP1 is a promising prognostic, therapeutic and immunological biomarker for pancreatic cancer: evidence from bioinformatics analysis and biological experiments

**DOI:** 10.1186/s12885-026-15685-0

**Published:** 2026-02-12

**Authors:** Shuhui Wang, Kaini He, Mimi Liu, Jiaxuan Zhou, Yujie Wen, Yan Cheng

**Affiliations:** Department of Digestive Disease and Gastrointestinal Motility Research Room, The Second Affiliated Hospital of Xi’an Jiaotong University, Shaanxi 710004 Xi’an, China

**Keywords:** Pancreatic cancer, MMP1, Prognosis, Immune infiltration

## Abstract

**Background:**

Pancreatic cancer remains one of the most lethal malignancies worldwide, underscoring the urgent need for reliable biomarkers to predict prognosis and guide therapy. This study aimed to identify and characterize such biomarkers using an integrated bioinformatics and experimental approach.

**Methods:**

The GEO database was used to screen genes differentially expressed in pancreatic cancer. Next, the DAVID database was used for GO functional enrichment and KEGG pathway analyses. STRING database and Cytoscape software were utilized to generate PPI networks and identify key genes. The prognostic value was then evaluated by utilizing the GEPIA database and Cox regression analysis. The relationship between key genes and clinicopathological parameters was analyzed using the UALCAN database. The TISCH2, TIMER, and TISDB databases were used for immune infiltration analysis. The functional role of the key gene was ultimately confirmed through in vitro experiments.

**Results:**

Through multiple rounds of strict screening, 10 key genes were obtained. After further verification, it was determined that MMP1 was significantly upregulated in pancreatic cancer tissue and correlated with poor prognosis in patients. Immunological analysis revealed correlations between MMP1 expression and the infiltration of specific immune cells (B cells, M1 macrophages, neutrophils, and monocytes) as well as the expression of immune checkpoint genes (VTCN1, LGALS9, TGFBR1, and IL10RB). In vitro functional assays confirmed that MMP1 knockdown in pancreatic cancer cells suppressed proliferation, migration, and invasion, while promoting apoptosis. Furthermore, a co-culture model demonstrated that MMP1 facilitated the recruitment of M1-polarized macrophages.

**Conclusion:**

MMP1 is an independent prognostic biomarker that drives pancreatic cancer progression through direct oncogenic effects and modulation of the tumor immune microenvironment. Thus, it emerges as an attractive, multifunctional therapeutic target, which calls for future research to confirm its translational potential and delineate the underlying molecular pathways.

## Introduction

Pancreatic cancer (PC) is one of the most deadly cancers in the world. According to statistics, PC ranks seventh among cance-related causes of death [1]. Its incidence rate is on the rise worldwide, and it is very likely to become the second leading cause of cancer death in Western countries [2]. PC initiates insidiously and advances swiftly, with the majority of patients presenting at an advanced stage upon diagnosis, resulting in a highly unfavorable prognosis. The 5-year survival rate stands at merely 11% [3]. Hence, it is imperative to identify and develop potential prognostic biomarkers and therapeutic targets.

In recent years, the integration of high-throughput microarray technology and bioinformatics has identified numerous genes implicated in cancer development. This approach enables a direct gene expression comparison across matched tumor and normal tissues, facilitating comprehensive analysis of differentially expressed genes (DEGs) and elucidation of tumorigenic pathways. Targeting such DEGs represents a viable strategy to inhibit cancer progression, as evidenced by PC research. For example, the DNA repair enzyme SMUG1 is upregulated in PC and enhances tumor proliferation, migration, and gemcitabine resistance, establishing it as a prognostic biomarker [4]. Meanwhile, the circular RNA Circ_0008768 suppresses tumor progression via the miR-330-3p/PTEN axis [5]. These studies underscore the therapeutic potential of modulating specific DEGs.

Over time, cancer has gradually been seen as a complex process with evolutionary and ecological characteristics, involving continuous, dynamic changes and close interactions between cancer cells and the tumor microenvironment (TME) [6]. Alterations in the TME contribute significantly to cancer progression. Within the TME, immune cells assume a central role in mediating cancer progression. An increasing amount of evidence suggests that the dynamic crosstalk between malignant and immune cells constitutes a key mechanism driving tumor progression and metastasis [7]. Within this context, members of the matrix metalloproteinase (MMP) family, key mediators of extracellular matrix (ECM) remodeling, are critically involved in facilitating tumor invasion, metastasis, and the dynamic modulation of the TME. For example, elevated MMP-2 expression is associated with poor prognosis in PC and represents an actionable therapeutic target, highlighting the broader significance of this protein family in oncology [8]. Despite revolutionizing the treatment landscape for several solid tumors, immune-oncology therapies have yielded limited success in PC [9]. Therefore, elucidating the distinct immune signature of PC is a critical endeavor.

In this study, we performed an integrated bioinformatics analysis using GEO datasets to identify DEGs in PC. Subsequent Gene Ontology (GO) and Kyoto Encyclopedia of Genes and Genomes (KEGG) pathway analyses, coupled with protein–protein interaction (PPI) network construction, identified ten hub genes. Among these, MMP1 was established as an independent prognostic risk factor, and its expression level showed significant correlation with key clinicopathological parameters in PC patients. Further immune profiling revealed associations between MMP1 expression, immune cell infiltration, and immune checkpoint molecules. Informed by the documented roles of other MMP family members [10, 11], we focused on characterizing the specific function of MMP1. Experimental validation confirmed that MMP1 was highly expressed in PC. Functional studies demonstrated that MMP1 knockdown suppressed cell proliferation, migration, and invasion, while inducing apoptosis. Additionally, MMP1 knockdown reduced the infiltration of M1 macrophages in an in vitro co-culture model. Collectively, our study identified MMP1 as a promising biomarker with integrated prognostic, therapeutic, and immunological relevance in PC.

## Materials and methods

### Data preparation and screening of DEGs

We downloaded GSE62452 and GSE183795 datasets from the Gene Expression Omnibus (GEO) database (https://www.ncbi.nlm.nih.gov/geo/) [12]. Among them, the GSE62452 dataset contained 69 PC tissues and 61 normal tissues; the GSE183795 dataset contained 139 PC tissues and 105 normal tissues. Subsequently, the GEO2R online analysis tool (https://www.ncbi.nlm.nih.gov/geo/geo2r/) was used to obtain DEGs, and the genes were rigorously screened based on the criteria of adj. *p*. val < 0.05 and |log2 fold change (FC)|> 1. The Volcano map was constructed to display the quantity and degree of DEGs, and the Venn diagram was utilized to exhibit the overlapping genes between the two datasets.

### GO function and KEGG pathway enrichment analyses

By utilizing the DAVID online tools (https://david.ncifcrf.gov/tools.jsp) [13], we revealed GO terms and KEGG pathways that were related to DEGs. GO analysis offered a comprehensive description of genes by examining their molecular functions, biological processes, and cellular components [14]. Additionally, we performed KEGG pathway analysis to elucidate the involved metabolic and signal transduction pathways [15].

### Construction of PPI network and selection of hub genes

STRING (https://cn.string-db.org/) is a comprehensive online analysis platform used for analyzing PPI [16]. We input DEGs into the STRING database, collected data from the PPI network, and visualized the network through Cytoscape software. Through the CytoHubba plugin in Cytoscape software, the Degree algorithm was used to analyze the correlation degree of DEGs in the PPI network, and the PPI level was used as the screening basis for key genes in PC. This study defined the top 10 genes with the highest PPI levels as key genes for the pathogenesis of PC.

### GEPIA (gene expression profiling interactive analysis)

GEPIA (http://gepia2.cancer-pku.cn/) is an online platform for comparative transcriptomics that leverages data from TCGA and GTEx. It enables systematic expression profiling between tumor and matched normal tissues across a large cohort (9,736 tumors and 8,587 normals). In addition, GEPIA can also perform survival analysis based on gene expression levels [17]. We used GEPIA to detect the expression level of hub genes in PC tissues and normal tissues, and drew survival curves to screen key genes related to the prognosis of PC.

### Clinicopathological parameters correlation with prognosis-related genes

The University of Alabama at Birmingham cancer data analysis portal (UALCAN) (http://ualcan.path.uab.edu/analysis.html) is a comprehensive, user-friendly, and interactive website aimed at providing users with convenient and in-depth TCGA gene expression data analysis services [18]. This study examined the expression of prognosis-associated genes both in PC versus normal tissues and across patient subgroups stratified by clinically relevant variables.

### Functional analysis and interaction network of similar genes

The UALCAN website was used to analyze the co-expressed genes with MMP1, and the top 20 genes in PC that were positively correlated with MMP1 expression were screened according to Pearson correlation coefficient. Metascape (https://metascape.org/gp/index.html) is a website that integrates multiple authoritative gene function annotation databases, aiming to provide users with comprehensive gene annotation and functional analysis [19]. We used the Metascape database to conduct pathway and process enrichment analyses of genes based on ontology sources such as KEGG pathway, GO Biological Processes, Reactome Gene Sets, Canonical Pathways, CORUM, and WikiPathways. GeneMANIA (http://www.genemania.org) is a data-rich, and user-friendly tool used to predict gene function and construct gene interaction networks. It integrates multiple data sources covering gene co-expression, PPIs, genetic interactions, biological pathways, and physical interactions, aiming to assist researchers in discovering other genes associated with specified genes [20]. Interaction networks among the similar genes were inferred using GeneMANIA.

### Immunoinfiltration analysis

Tumor Immune Single-cell Hub 2 (TISCH2) (http://tisch.comp-genomics.org/) is an online platform dedicated to tumor scRNA seq data. The platform provides detailed cell type labeling at the single-cell level, making it easy for users to explore the microenvironment of various types of tumors [21]. In this study, we used six datasets (PAAD_CRA001160, PAAD_GSE111672, PAAD_GSE154763, PAAD_GSE154778, PAAD_GSE158356, and PAAD_GSE165399) to analyze the expression of MMP1 in different cell types of PC**.** TIMER2.0 (http://timer.cistrome.org/) is an online tool designed to systematically analyze the immune infiltration status of different types of cancer. It consists of three major components: immune, exploration, and estimation. Based on the expression profile data provided by users, the evaluation component can reliably assess the level of immune cell infiltration using six advanced algorithms [22]. Using TIMER2.0, we assessed correlations of MMP1 expression with both immune infiltration and immune checkpoint levels in PC.

### Tissue microarray

PC tissue microarray was sourced from Outdo Biotech Co., Ltd (Shanghai, China). The chip array number was HPanA030PG04, which included 23 cases of PC tissues and 7 cases of adjacent non‐malignant tissues.

### Cell lines and transfection

PC (PANC-1, SW1990) and normal pancreatic ductal epithelial (HPDE) cell lines were obtained from the Cell Bank of the Chinese Academy of Sciences. SiRNAs (si-NC, si-MMP1) were purchased from Tsingke Biotechnology Co., Ltd (Shanghai, China). Transfection of siRNAs into PC cells was carried out with Lipofectamine 3000.

### Western blotting analysis

Firstly, total protein was extracted from the cells using RIPA buffer and protease inhibitors. We separated proteins by SDS-PAGE electrophoresis, and then transferred the separated proteins onto a PVDF membrane. Next, we blocked the membrane with 10% skim milk and incubated with primary antibodies (MMP1, Proteintech; β-actin, Proteintech) at 4 °C overnight. We washed the PVDF membrane and incubated with the corresponding secondary antibody. Finally, protein bands were exposed and visualized using an ECL chemiluminescence assay kit and a chemiluminescence imaging instrument.

### CCK8 assay

Cell viability was assessed at 24, 48, 72, and 96 h using the Cell Counting Kit-8 (UElandy) according to the manufacturer’s instructions.

### Wound healing assay

Cells were seeded in a 6-well plate. Upon reaching confluence, a straight scratch was created using a sterile pipette tip. After discarding the old medium and washing the cells, serum-free medium was added. Wound images were captured at 0 and 48 h under a microscope.

### Transwell migration and invasion assays

Migration assay: Cells were seeded in serum-free medium into the upper Transwell chamber, with the lower chamber containing DMEM supplemented with 30% FBS. After 24 h, non-migrated cells were removed from the upper membrane by swabbing. Migrated cells on the lower surface were then fixed with methanol, stained with crystal violet, and imaged.

Invasion assay: The procedure for the invasion assay was similar to the migration assay, with the critical modification that the Transwell membrane was pre-coated with Matrigel (Corning, USA) to simulate the ECM barrier. Briefly, Matrigel was diluted in cold serum-free medium and uniformly coated onto the upper chamber membrane, followed by polymerization at 37 °C for 6 h. Subsequently, digested cells were seeded onto the solidified Matrigel layer. The subsequent steps of incubation, fixation, staining, and quantification were identical to those described for the migration assay.

### Co-culture of M1 macrophages and PC cells

To investigate the role of MMP1 in macrophage recruitment, a Transwell co-culture system was established. Briefly, RAW264.7 murine macrophage cells were treated with LPS (1 μg/mL, 24 h) to achieve M1 polarization, which was confirmed by the upregulation of the surface marker CD80 via flow cytometry. Subsequently, LPS-induced M1 macrophages were placed in the upper chamber, and co-cultured with PC cells in the lower chamber. After 24 h of co-culture, non-migratory cells were cleared from the upper membrane; migrated macrophages on the lower side were then fixed, stained with crystal violet, and quantified.

### Statistical analysis

SPSS statistics 18.0 and GraphPad Prism 8 were uesd to analyze the data. The impacts of variables on prognosis were analysed by univariate and multivariate Cox proportional hazards modelling. One-way ANOVA was used to compare multiple groups. Data were reported as means ± SD, and *p* ≤ 0.05 was considered statistically significant.

## Results

### Identification of DEGs

**Fig. 1:**
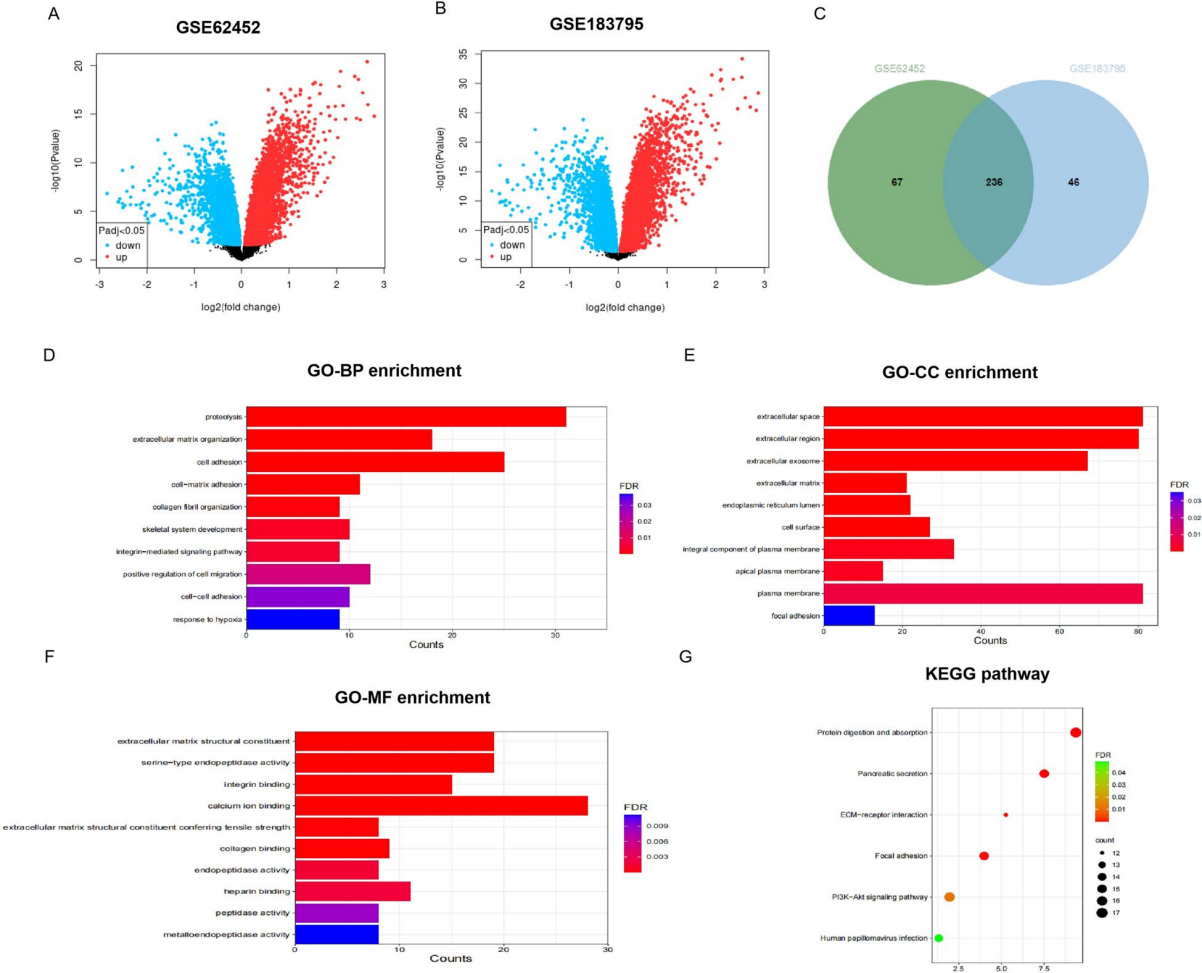
Identification and Enrichment Analysis of differentially expressed gene (DEGs). **A** Volcano plot of DEGs obtained from the GSE62452 dataset. **B** Volcano plot of DEGs obtained from the GSE183795 dataset. **C** Venn diagram of DEGs. **D**–**F** Top 10 GO term enrichment analysis of DEGs: **D** GO-biological process terms. **E** GO-cellular component terms. **F** GO-molecular function terms. **G** KEGG pathway enrichment analysis of DEGs

Using adj. *p*. val < 0.05 and |log2FC| > 1 as criteria, 303 DEGs were selected from 33,297 genes in the GSE62452 dataset, and 282 DEGs were selected from 19,246 genes in the GSE183795 dataset. The volcano plots of DEGs from two datasets were shown in Fig. 1A-B. Intersected the DEGs of two datasets and selected 236 common DEGs. The Venn diagram was shown in Fig. 1C.

### Enrichment analysis of DEGs

Functional enrichment analysis was performed on the overlapping DEGs using DAVID. GO analysis revealed that BP enrichment mainly occurred in proteolysis, ECM organization, and cell adhesion; CC terms were mainly enriched in extracellular space and extracellular region; MF terms were most highly enriched in ECM structural constituent, serine−type endopeptidase activity, and calcium ion binding (Fig. 1D-F). KEGG analysis further identified protein digestion and absorption as well as pancreatic secretion as the most significantly enriched pathways (Fig. 1G).

### PPI network construction

**Fig. 2:**
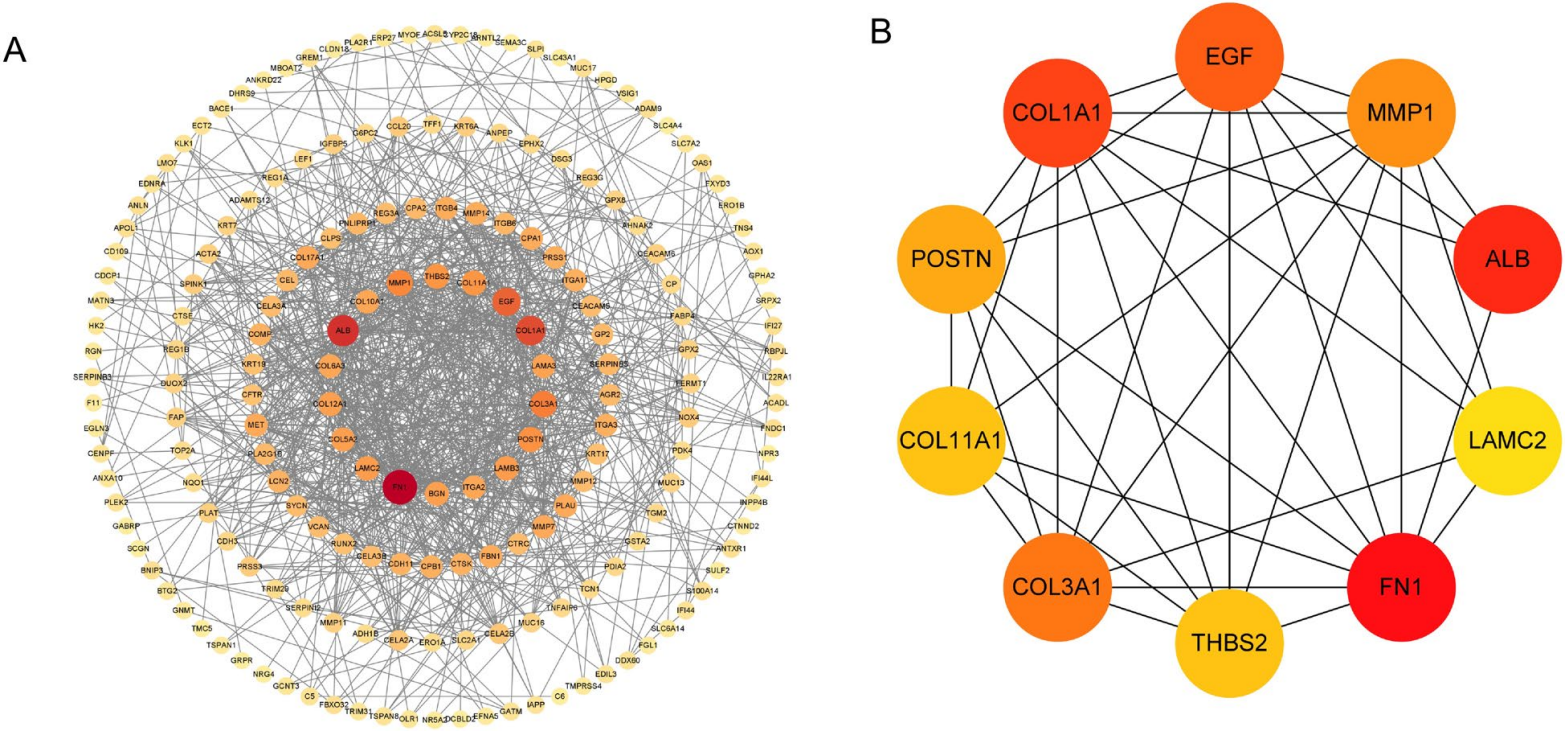
Construction of PPI network and selection of hub genes. **A** The PPI networks for differentially expressed genes. **B** The 10 hub genes in the PPI network

Using the STRING database and the Degree algorithm of the CytoHubba plugin in Cytoscape software, we constructed a PPI network for the DEGs and screened the top 10 hub genes: FN1, ALB, COL1A1, EGF, COL3A1, MMP1, POSTN, COL11A1, THBS2, and LAMC2 (Fig. 2).

### Gene expression of the hub genes from the GEPIA database

**Fig. 3:**
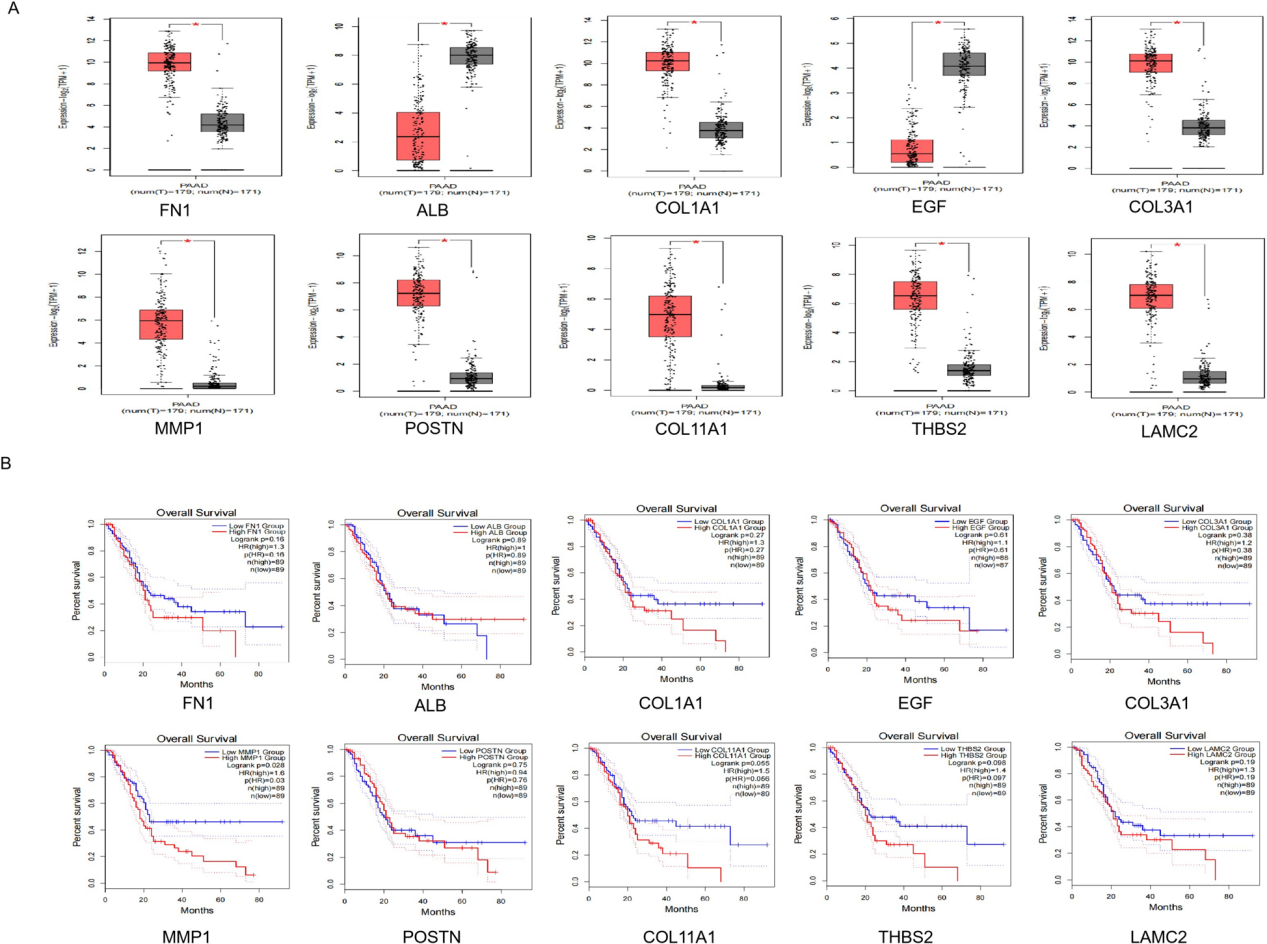
Expression and survival analysis of 10 hub genes in PC. **A** GEPIA showed the expression of 10 hub genes in PC tissues and normal pancreatic tissues (*p < 0.05). **B** Survival analysis of 10 hub genes in PC using GEPIA. *HR* Hazard ratio

The expression level of 10 hub genes was confirmed by using the GEPIA dataset. Among them, 8 genes (FN1**,** COL1A1**,** COL3A1**,** MMP1**,** POSTN**,** COL11A1**,** THBS2**,** LAMC2) were upregulated in PC tissues, and 2 genes (ALB**,** EGF) were downregulated in PC tissues. The differences were statistically significant (Fig. 3A). These results were also consistent with what we detected in the GEO database.

### Screening for prognosis-related genes

Survival analysis using GEPIA2.0 revealed that among the 10 hub genes, only MMP1 expression was significantly correlated with overall survival in PC patients, with lower expression predicting a better prognosis. The remaining 9 genes showed no significant association (Fig. 3B).

### MMP1 could be an independent risk factor for predicting the prognosis of PC

Clinical data from 178 patients with PC in TCGA database were analyzed to evaluate MMP1 as an independent prognostic factor. Following the exclusion of cases with incomplete records, univariate Cox regression identified high MMP1 expression as a risk factor for poor survival. Additionally, clinical parameters including T classification and N classification were also related to the prognosis of PC patients (Table 1). Multivariate analysis further established MMP1 as a potential independent prognostic biomarker (Table 2).

**Table 1:** Prognostic factors in PC patients by univariate analysis

Variables	Risk ratio	95% confidence interval	*p*
Age	1.019	0.997–1.042	0.084
Gender	0.924	0.598–1.430	0.723
Grade (G1-G2 vs. G3-G4)	1.568	0.977–2.518	0.063
Stage (Ⅰ-Ⅱ vs. Ⅲ-Ⅳ)	0.776	0.244–2.467	0.667
T (T1-T2 vs. T3-T4)	1.968	1.032–3.750	0.040
N (N0 vs. N1)	1.922	1.122–3.293	0.017
M (M0 vs. M1)	1.086	0.258–4.574	0.911
MMP1	1.155	1.062–1.256	0.001

**Table 2:** Multivariate analysis using the Cox proportional hazards model

Variables	Risk ratio	95% confidence interval	*p*
Age	1.018	0.996–1.040	0.114
Grade (G1-G2 vs. G3-G4)	1.436	0.880–2.344	0.148
Stage (Ⅰ-Ⅱ vs. Ⅲ-Ⅳ)	0.886	0.269–2.921	0.842
T (T1-T2 vs. T3-T4)	1.295	0.638–2.628	0.474
N (N0 vs. N1)	1.536	0.843–2.799	0.161
MMP1	1.118	1.014–1.232	0.026

### Correlation of MMP1 expression with clinicopathological features in PC patients

**Fig. 4:**
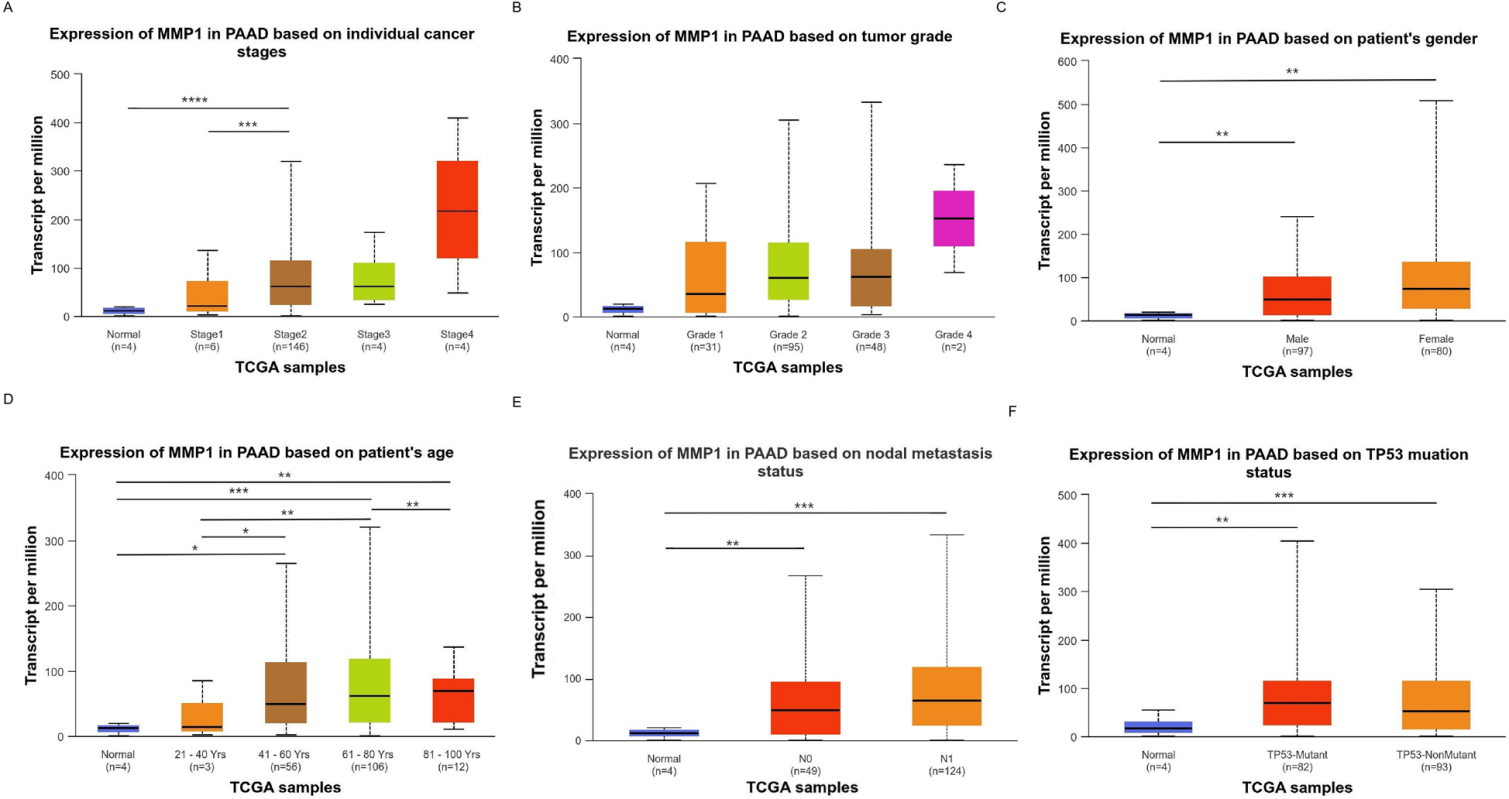
Correlation of MMP1 expression with clinicopathological features in PC patients (UALCAN database). Expression of MMP1 in PC patients based on different (**A**) stages, (**B**) grades, (**C**) genders, (**D**) ages, (**E**) lymph node metastasis status, and (**F**) TP53 mutation status. *p < 0.05, **p < 0.01, ***p < 0.001, ****p < 0.0001

Next, UALCAN database analysis showed that MMP1 expression in PC was associated with cancer stage (specifically, higher in Stage 2 vs. Stage 1) and patient age. No significant variation in MMP1 levels was observed across other clinicopathological parameters, including tumor grade, gender, nodal status, or TP53 mutation status (Fig. 4A-F).

### Functional analysis and interaction network of genes related to MMP1 in PC

**Fig. 5:**
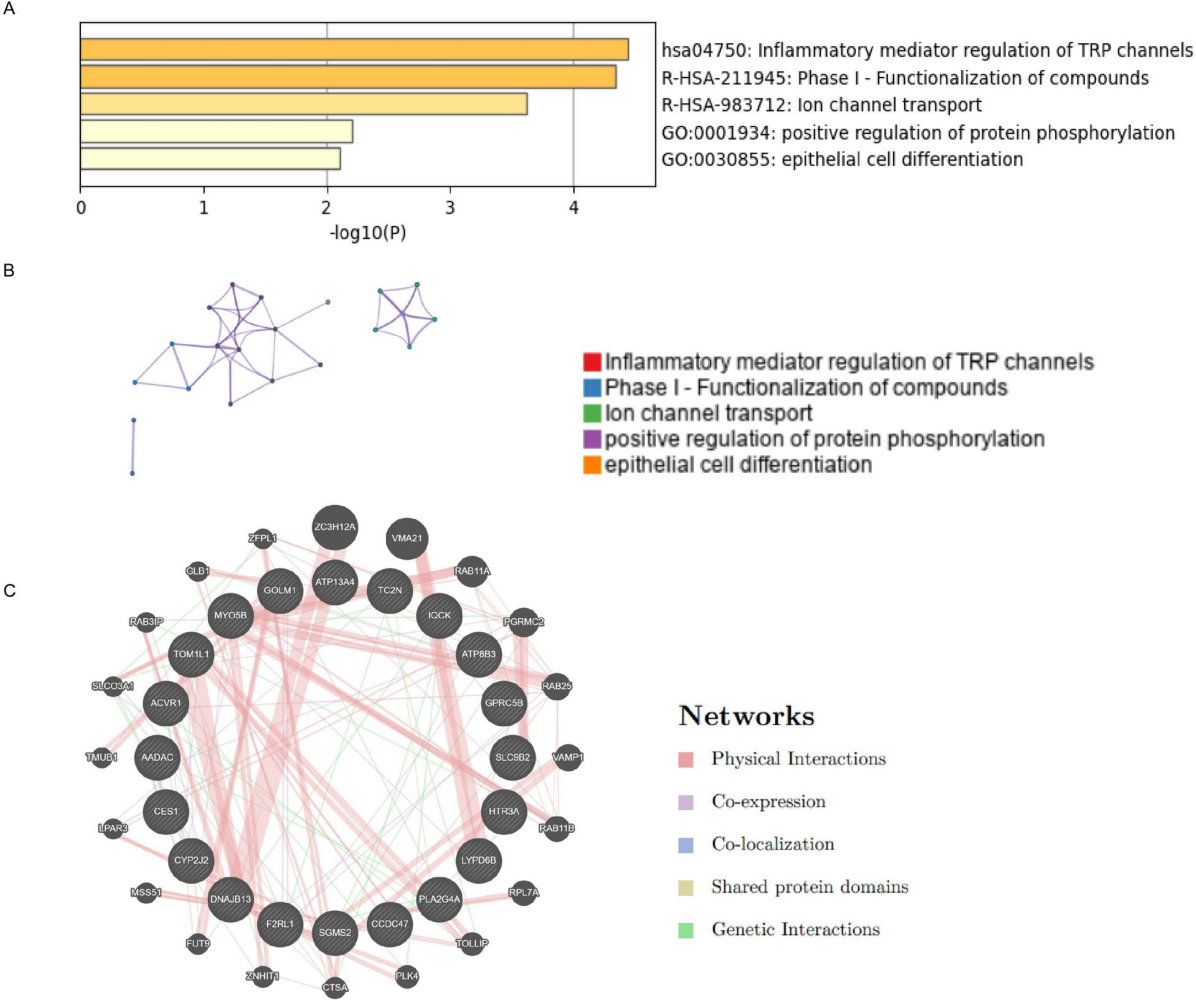
Functional analysis and interaction network of genes related to MMP1 in PC. **A** Bar graph of enriched terms across the top 20 genes. **B** Network of enriched terms colored by cluster ID. **C** Top 20 genes and their co-expression genes

To investigate MMP1's functional network in PC, we first identified its top 20 Pearson-correlated genes using UALCAN. Metascape enrichment analysis revealed their primary roles in inflammatory mediator regulation of transient receptor potential (TRP) channels, phase Ⅰ-functionalization of compounds, ion channel transport, positive regulation of protein phosphorylation, and epithelial cell differentiation (Fig. 5A-B). A subsequent GeneMANIA network analysis indicated that physical interactions (52.25%) and co-expression (42.24%) were the predominant connection types among these genes (Fig. 5C).

### Correlation of MMP1 expression with immunoinfiltration and immune checkpoints

**Fig. 6:**
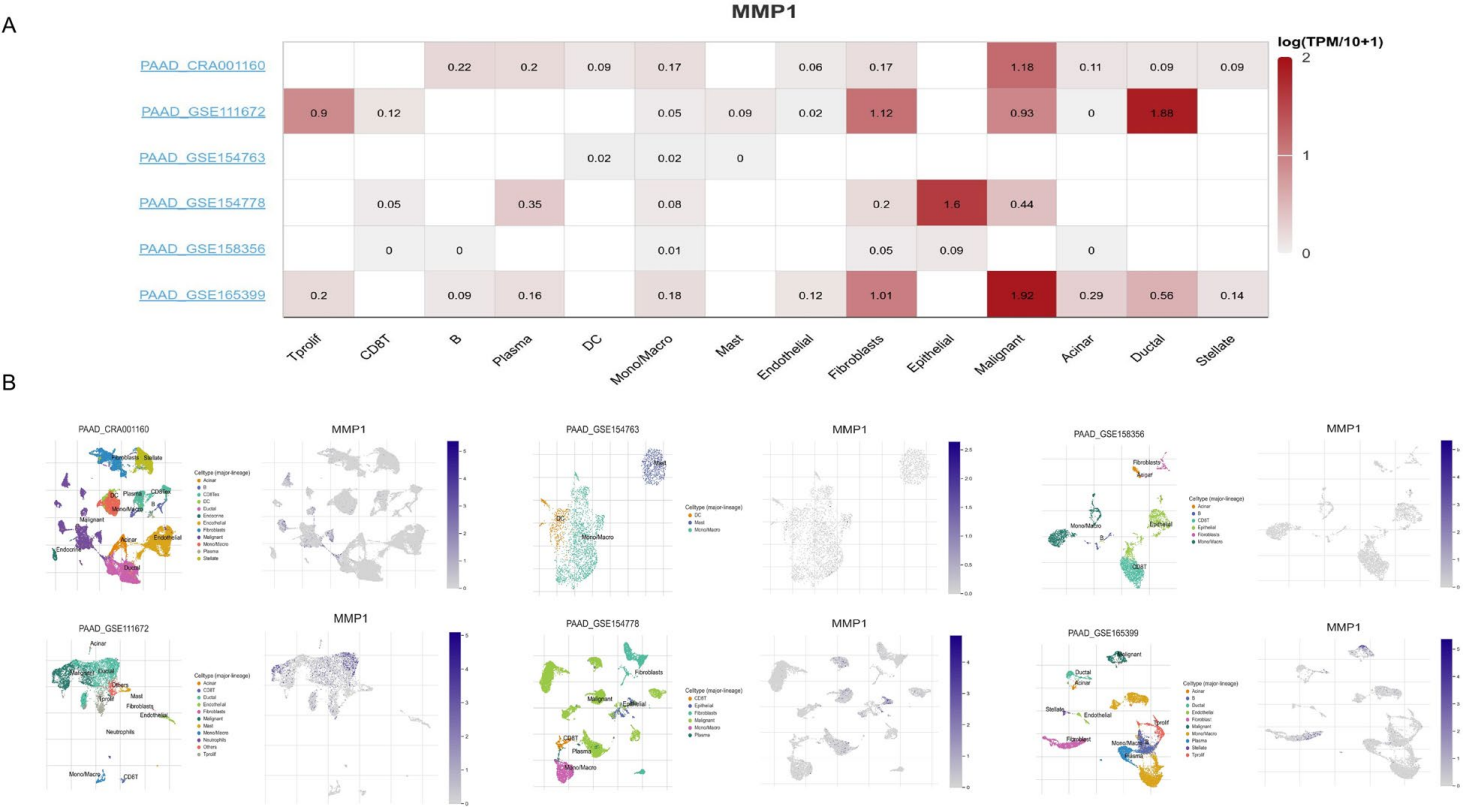
MMP1 expression in TME-related cells. **A** The heatmap displayed the average expression level of MMP1 in TME-related cells of PC. **B** Expression of MMP1 at single-cell resolution in the PC datasets

**Fig. 7:**
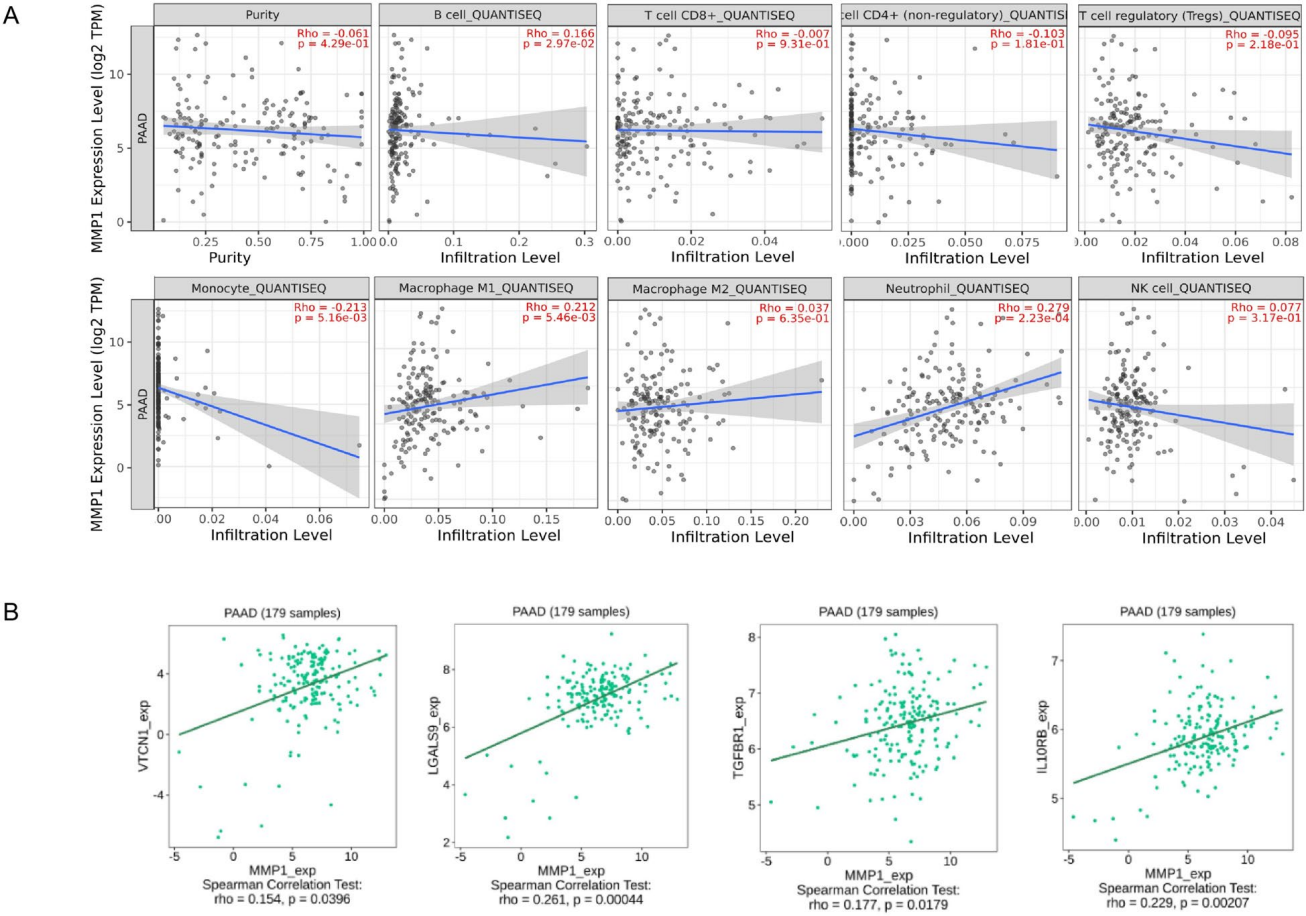
Correlation of MMP1 expression with immunoinfiltration and immune checkpoints. **A** Correlation between MMP1 expression with immune cell infiltration. **B** Correlation between MMP1 expression with immune checkpoints

Given the established link between cancer and the immune microenvironment, we hypothesized that MMP1 might influence pancreatic cancer (PC) progression via immunomodulation. Analysis of single-cell sequencing data from the TISCH database revealed that MMP1 was predominantly highly expressed in malignant, ductal, and epithelial cells, but showed low expression in immune cells (Fig. 6). Subsequent evaluation using the QUANTISEQ algorithm from the TIMER2.0 database indicated that MMP1 expression levels correlated positively with the infiltration of B cells, M1 macrophages, and neutrophils, and negatively with monocyte infiltration in PC (Fig. 7A). Furthermore, MMP1 expression showed significant positive correlations with the immune checkpoint molecules VTCN1, LGALS9, TGFBR1, and IL10RB (Fig. 7B). Collectively, these findings suggested that MMP1 might affect the progression of PC through immune escape and could be used as a therapeutic target.

### The expression of MMP1 in PC and its effect on tumor cell proliferation, apoptosis, migration, and invasion

**Fig. 8:**
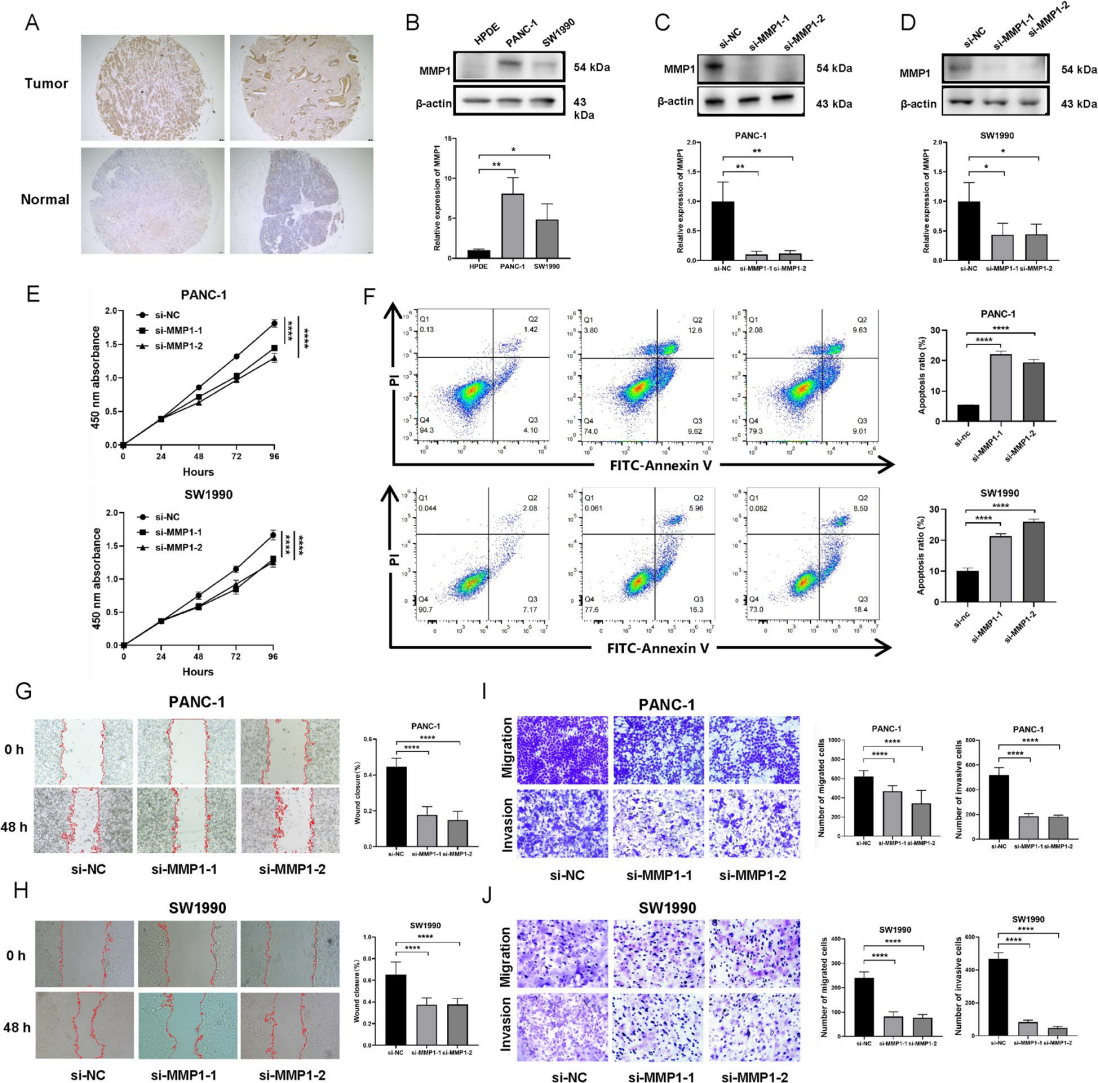
The expression of MMP1 in PC and its effect on the proliferation and migration of PC cells. **A** Representative images of immunohistochemical staining of MMP1 expression in PC tissues and adjacent non‐tumour tissues. **B** Detection of MMP1 protein levels in cell lines using Western blotting. **C**-**D** MMP1 knockdown was confirmed by Western blotting. **E** CCK-8 assay showed that knocking down the expression of MMP1 significantly inhibited cell proliferation. **F** Flow cytometric analysis indicated that MMP1 silencing promoted apoptosis. **G**-**H** Wound healing assay showed that the migratory ability was significantly reduced in the MMP1‐knockdown PANC-1 (**G**) and SW1990 (**H**) cells. **I**-**J** Transwell migration and invasion assays consistently showed that MMP1 knockdown reduced both the migratory and invasive capacities of PANC-1 (**I**) and SW1990 (**J**) cells. All the data were expressed as the means ± SD of three independent experiments. *p < 0.05, **p < 0.01, ****p < 0.0001

Building on the association between MMP1 and both prognosis and immune infiltration in PC, we next sought to investigate its functional role. Immunohistochemical analysis of a PC tissue microarray revealed significantly higher MMP1 expression in tumor tissues compared to adjacent non-tumorous tissues (Fig. 8A). Consistent with this, MMP1 expression was markedly elevated in PC cell lines (PANC-1 and SW1990) relative to the normal pancreatic ductal epithelial cell line HPDE (Fig. 8B). To investigate the tumor-promoting function of MMP1, we transfected siRNAs targeting MMP1 into PANC-1 and SW1990 cells. Western blotting confirmed efficient knockdown of MMP1 protein in both cell lines (Fig. 8C-D). Subsequent functional experiments were performed at time points established in standard protocols [23, 24]. To investigate the role of MMP1 in proliferation, CCK-8 assays were performed. Relative to si-NC controls, MMP1 knockdown led to significant growth inhibition in PANC-1 and SW1990 cells (Fig. 8E). Apoptosis analysis by flow cytometry confirmed that MMP1 silencing promoted cell death (Fig. 8F). Additionally, migration was determined using wound healing and Transwell assays. The data indicated that MMP1 knockdown substantially attenuated the migratory ability of PANC-1 and SW1990 cells (Fig. 8G-J). Finally, to evaluate the invasive capacity, a Transwell invasion assay was conducted using chambers coated with Matrigel to simulate the ECM environment. Silencing MMP1 significantly reduced the invasive potential of PC cells (Fig. 8I-J).

### MMP1 promotes the recruitment of M1 macrophages in a PC co-culture model

**Fig.9:**
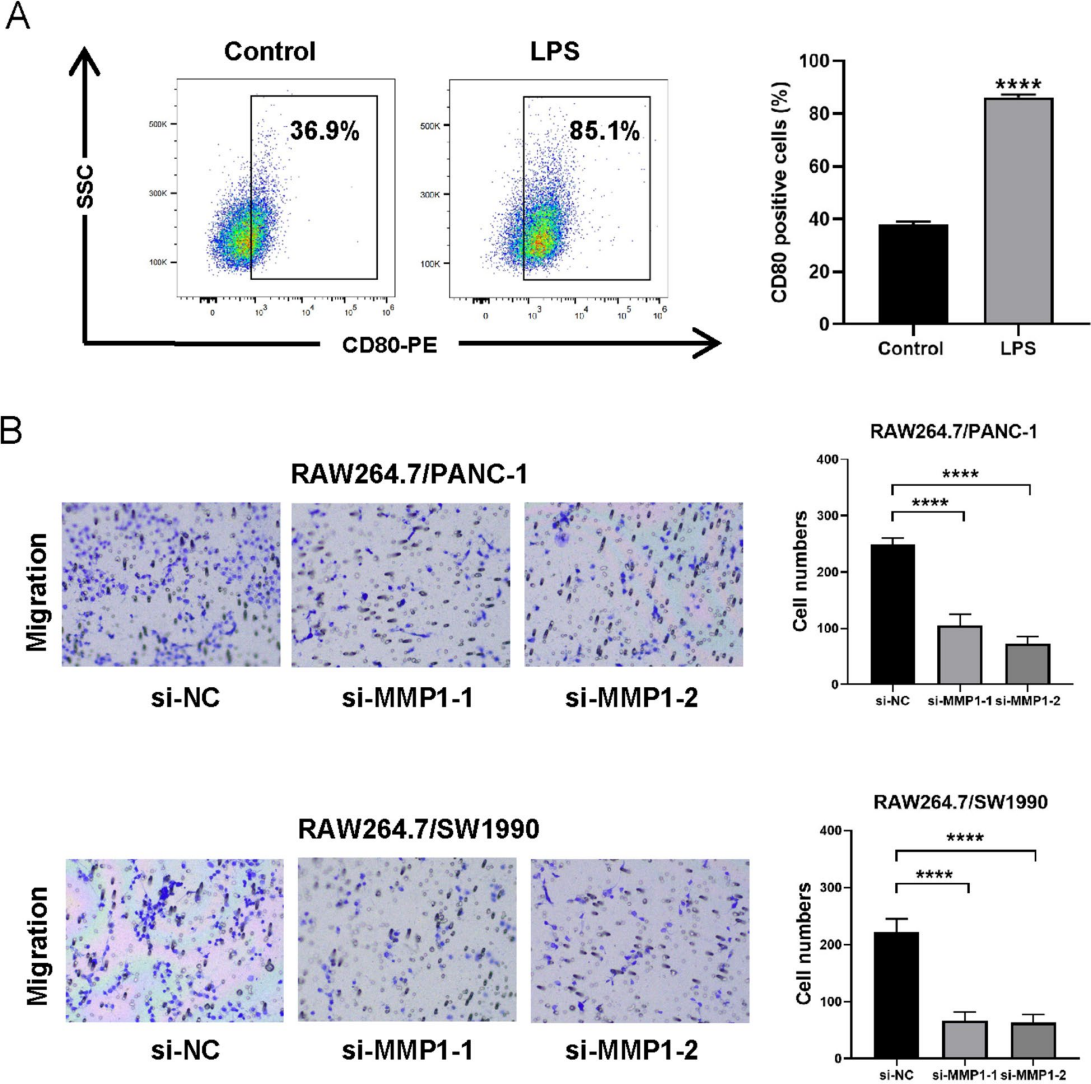
MMP1 promotes the recruitment of M1 macrophages in a PC co-culture model. **A** Flow cytometric analysis confirms LPS-induced M1 polarization in RAW264.7 cells. **B** MMP1 knockdown attenuates M1 macrophage migration in a co-culture model. ****p < 0.0001

Macrophages are pivotal regulators within the TME, and their polarization states critically influence cancer progression. Notably, Xu et al. reported that MMP1 promotes the infiltration of M1-type macrophages [25], which aligns with our bioinformatic predictions. To validate this functional association in PC, we established a co-culture model. RAW264.7 cells were polarized to the M1 phenotype by LPS stimulation, as confirmed by the upregulation of CD80 via flow cytometry (Fig. 9A). To assess macrophage recruitment, LPS-induced M1 macrophages were co-cultured with si-MMP1 or si-NC-transfected PANC-1/SW1990 cells in a Transwell system for 24 h, followed by quantification of migrated macrophages. MMP1 knockdown in both cancer cell lines significantly reduced M1 macrophage migration compared to the si-NC groups (Fig. 9B). This finding demonstrated that MMP1 facilitated the recruitment of M1-polarized macrophages within the TME.

## Discussion

The changes in gene transcription levels are an important driving force for cancer development, affecting tumor formation, progression, and metastasis. Public databases such as GEO and TCGA cover a wide range of gene expression data and clinical information, providing strong support for in-depth exploration of tumor biological mechanisms. Our analysis of GEO datasets uncovered 236 common DEGs in PC. Subsequently, GO and KEGG analyses showed that the biological processes of DEGs were related to ECM and protein digestion and absorption. The ECM, a central element of the TME, not only provides mechanical support for cells but also acts as a repository of signaling molecules. Increased ECM stiffness is known to impede immune cell activation, migration, and infiltration, thereby fostering tumor progression [26]. Consequently, the ECM has emerged as a promising therapeutic target in oncology [27, 28].

In this study, PPI network analysis identified FN1, ALB, COL1A1, EGF, COL3A1, MMP1, POSTN, COL11A1, THBS2, and LAMC2 as potential key genes in PC development. Survival analysis revealed MMP1 as the only gene among these genes significantly associated with patient prognosis. MMPs, as a family of endopeptidases, can degrade almost all components of the ECM and regulate various cellular behaviors associated with cancer, such as invasion, metastasis, and immune escape [29]. Mounting evidence underscores the critical functions of MMPs across various stages of cancer [30]. Notably, MMP1 (a principal MMP) is often overexpressed in tumors and associated with poor survival, including colon cancer [31], breast cancer [32], cervical squamous cell carcinoma [33]. While its role in PC remains incompletely understood, our data demonstrated that MMP1 was upregulated in PC, and its elevated expression was associated with significantly worse survival. Furthermore, Cox regression analysis further validated its role as a standalone prognostic indicator, with its expression levels also tied to both tumor stage and patient age. However, a limitation of this study is that detailed treatment records were unavailable in the public datasets used, precluding adjustment for therapy-related confounders. Thus, prospective studies with comprehensive clinical data are needed to confirm the independent prognostic value of MMP1. Moreover, mechanistic research indicates that knockdown of the E26 transformation specific sequence-1 (Ets-1) restores gemcitabine sensitivity while downregulating MMP1 [34], suggesting a potential role for MMP1 in chemoresistance. Taken together, these findings identify MMP1 as a prognostic biomarker for PC and as a potential target for combination chemotherapy, although this potential requires rigorous future validation. Future studies could analyze efficacy data from clinical cohorts or establish drug-resistant cell models for in vitro validation, thereby providing more direct evidence for MMP1 as a combinatorial therapeutic target.

At the functional level, our in vitro experiments yielded findings consistent with an oncogenic role for MMP1. Efficient knockdown of MMP1 was achieved in both PANC-1 and SW1990 cell lines. Silencing MMP1 significantly inhibited cell proliferation, induced apoptosis, and impaired migratory and invasive capacities in these models, suggesting its pro-tumorigenic function.

To further elucidate the role of MMP1 in PC, we identified genes with potential synergistic functions. Analysis revealed their primary enrichment in pathways regulating inflammatory mediators within TRP channels—a major class of Ca^2+^-permeable ion channels that modulate intracellular signaling and cellular function by controlling cation fluxes. Accumulating evidence has elucidated the important role of TRP channels in the occurrence and development of tumors, and TRP channels have become targets for studying tumor biological behavior and exploring new treatment strategies [35]. Recent studies indicate an upregulation of TRP channels in PC, which correlates with unfavorable tumor outcomes and disease progression‌ [36, 37]. In addition, studies have revealed that TRPM7, a member of the TRPM family, could regulate the expression of MMP2 and participate in the development of PC and glioblastoma [38, 39]. These findings point to a role for TRP channels in PC progression via MMP1 regulation, which needs further experimental verification.

Infiltrating immune cells represent an integral component of the solid tumor microenvironment and shape tumor progression [40]. However, the impact of MMP1 expression on immune cell recruitment is still unclear. Analysis using the TIMER2.0 database and QUANTISEQ algorithm revealed that MMP1 expression in PC correlated with the infiltration levels of B cells, M1 macrophages, neutrophils, and monocytes. Importantly, our in vitro co-culture model provided experimental validation, demonstrating that knockdown of MMP1 in PC cells attenuated the recruitment of M1-polarized macrophages. Although classically activated M1 macrophages are generally considered to possess anti-tumor properties, emerging evidence reveals a more complex scenario in PC. Studies indicate that in PC, both exogenously supplied M1-like and M2-like macrophages can drive metastasis. Furthermore, PC cells can reprogram M1-like macrophages toward a pro-tumorigenic fate through mechanisms involving GARP and DNA methylation [41]. This suggests that the pro-tumor effect observed upon MMP1-mediated M1 macrophage recruitment may stem from the unique, immune-suppressive TME of PC, which reshapes macrophage function. The specific mechanisms by which tumor cells reprogram M1-like macrophages merit future investigation and hold high potential for identifying novel therapeutic targets.

The limited efficacy of immunotherapy in PC is closely associated with its immunosuppressive TME [42–44]. Our findings reveal a positive correlation between MMP1 expression and key immune checkpoint molecules, including VTCN1, LGALS9, TGFBR1, and IL10RB. This correlation suggests that MMP1 may contribute to immune evasion beyond its canonical role in ECM remodeling, aligning with broader analyses that link MMP1 to adverse immune profiles and poor prognosis. The specific checkpoints co-expressed with MMP1 are each implicated in pivotal and distinct immunosuppressive pathways within PC. For instance, VTCN1 operates by binding to its receptor on antigen-presenting cells, thereby inhibiting T cell activation [45]. LGALS9 contributes to immune suppression by driving T cell apoptosis and programming macrophages toward a tolerogenic phenotype [46]. TGFBR1 mediates TGF-β signaling, which suppresses effector T cell function and promotes regulatory T cell expansion [47]. Furthermore, IL10RB is a central component of the IL-10 receptor, mediating signaling that drives T cell exhaustion [48], a hallmark of the PC microenvironment. Thus, MMP1 might function as a critical regulatory node that connects tumor invasiveness to the coordinated expression of multiple immune-inhibitory pathways, potentially licensing a broadly resistant microenvironment. Future studies should aim to clarify how MMP1 regulates these checkpoints, elucidating the underlying molecular mechanisms to identify new targets for PC combination immunotherapy.

Despite the progress made, several shortcomings of this study must be noted. First, our findings on MMP1 expression and its prognostic value are primarily based on public databases and a PC tissue microarray. The lack of validation in a large, multi-center clinical cohort limits the generalizability and reliability of these results. Second, while we demonstrated that MMP1 promotes the recruitment of M1 macrophages, the precise molecular mechanisms underlying this process remain unclear and warrant further investigation. Furthermore, future studies should develop more complex in vitro or in vivo models to systematically elucidate whether and how MMP1 influences the composition and functional states of broader immune cell communities within the tumor microenvironment. Third, although we observed correlations between MMP1 and immune checkpoint genes, the molecular pathways through which MMP1 regulates these checkpoints were not explored. Future studies incorporating pathway analyses or interaction experiments are needed to elucidate how MMP1 modulates immune checkpoint expression and function in PC.

In summary, this study demonstrated that elevated MMP1 expression was associated with unfavorable clinical outcomes in patients with PC. MMP1 was upregulated in both PC tissues and cell lines, in which it promoted cellular proliferation, migration, invasion, and resistance to apoptosis. Using a co-culture model, we further confirmed that MMP1 facilitated the migration of M1 macrophages toward tumor cells. Additionally, the expression of MMP1 was closely linked to both the infiltration of specific immune cells and the expression of immune checkpoint markers, suggesting its involvement in remodeling the tumor immune microenvironment. These findings position MMP1 as a promising dual-role target in PC, serving as both a prognostic biomarker and a potential therapeutic target. Future studies are warranted to validate its prognostic value in larger clinical cohorts and to further elucidate its mechanisms in chemoresistance and immune regulation.

## Conclusion

This study identifies MMP1 as a key driver of PC progression, serving as an independent prognostic biomarker. MMP1 contributes to tumor progression not only by promoting cellular proliferation, migration, invasion, and apoptosis resistance but also by remodeling the tumor immune microenvironment via the recruitment of M1 macrophages, and it is additionally correlated with immune checkpoint molecule expression. Collectively, this evidence highlights MMP1 as a promising therapeutic target with multiple points of intervention, warranting future investigation to fully elucidate its mechanisms and translational potential.

## Data Availability

The datasets analysed during the current study are available in the GEO database(https://www.ncbi.nlm.nih.gov/geo/). All data are included in this article and are also available from the corresponding author on reasonable request.
